# The Potential Role of Nutrition in Lung Cancer Establishment and Progression

**DOI:** 10.3390/life12020270

**Published:** 2022-02-12

**Authors:** Chiara Porro, Maria Ester La Torre, Nicola Tartaglia, Tarek Benameur, Mario Santini, Antonio Ambrosi, Giovanni Messina, Giuseppe Cibelli, Alfonso Fiorelli, Rita Polito, Gaetana Messina

**Affiliations:** 1 Department of Clinical and Experimental Medicine, University of Foggia, 71122 Foggia, Italy; chiara.porro@unifg.it (C.P.); ester.latorre@unifg.it (M.E.L.T.); giovanni.messina@unifg.it (G.M.); giuseppe.cibelli@unifg.it (G.C.); 2 Department of Medical Additionally, Surgical Sciences, University of Foggia, 71100 Foggia, Italy; nicola.tartaglia@unifg.it (N.T.); antonio.ambrosi@unifg.it (A.A.); 3 Department of Biomedical Sciences, College of Medicine, King Faisal University, Al-Ahsa 31982, Saudi Arabia; tbenameur@kfu.edu.sa; 4 Department of Translational Medicine, Università degli Studi della Campania “Luigi Vanvitelli”, 80138 Naples, Italy; mario.santini@unicampania.it (M.S.); gaetana.messina@unicampania.it (G.M.)

**Keywords:** lung cancer, obesity, healthy lifestyle, correct nutrition, oxidative stress, inflammation

## Abstract

Lung cancer is a devastating disease with a high incidence and low survival rates, so recent studies have focused on analyzing the risk factors that might prevent this disease from developing or have protective/therapeutic effects. Nutrition is an important key factor in the prevention and treatment of lung cancer. Various factors appear to be involved in the development of the latter, such as cigarette smoking or certain external environmental factors. The increase in oxidative stress is therefore an integral part of the carcinogenesis process. The biological role of bioactive factors derived from adipose tissue, mainly adipokines, is implicated in various cancers, and an increasing body of evidence has shown that certain adipocytokines contribute to the development, progression and prognosis of lung cancer. Not all adipokines stimulate tumor growth; in fact, adiponectin inhibits carcinogenesis by regulating both cell growth and the levels of inflammatory cytokines. Adiponectin expression is deregulated in several cancer types. Many nutritional factors have been shown to increase adiponectin levels and therefore could be used as a new therapeutic strategy for combating lung cancer. In addition, foods with antioxidant and anti-inflammatory properties play a key role in the prevention of many human diseases, including lung cancer. The purpose of this review is to analyze the role of diet in lung cancer in order to recommend dietary habit and lifestyle changes to prevent or treat this pathology.

## 1. Introduction

Lung cancer (LC) remains the leading cause of cancer deaths worldwide, with 14.1 million new cancer cases and 8.2 million deaths annually [1]. The global incidence appears to be variable but evenly distributed, with an upward trend among males and females [2].

The incidence rate increases with age [3], and the predisposition is stronger in men than in women [4]. Ethnicity is also an important factor for genetic predisposition. In fact, black men are 20% more likely to develop lung cancer than white men, but at the same time 30% less likely to develop small-cell lung cancer [5].

Lung cancer is divided into two main subtypes, small cell lung cancer (SCLC) and non-small cell lung cancer (NSCLC), the latter accounting for 85–90% of all LC cases [6]. About 84% correspond to all of lung cancer diagnoses [4]. Beyond this first division, they can be divided into four main histological subtypes: adenocarcinoma, squamous-cell carcinoma, small-cell carcinoma and large-cell carcinoma. Adenocarcinoma, squamous-cell carcinoma and large-cell carcinoma belong to the first subtype [4]. Small-cell LC has been found to be the deadliest as it causes more metastases [7].

Unfortunately, nearly 57% of lung cancers have already metastasized by the time they are diagnosed, resulting in a five-year survival rate for about 4.7% of the total cases. In addition, only about 18.6% of diagnosed people survive after 5 years [8].

The response to current cytotoxic therapies has reached a plateau in terms of response rate and survival [6]. For years, this pathology has been considered as a monofactorial disease, but recently, due to the heterogeneity of this pathology, it is believed that lung cancer is characterized by several phenotypes, rendering it a complex syndrome. A close association between pulmonary epithelium and adipose tissue has recently been identified, since the secretion of adipokines, including adiponectin, is altered in many pathological conditions of the lungs [9].

Adipose tissue is the main deposit of triglycerides in mammals, including humans. It is made up of several cells called adipocytes, which are responsible for the synthesis and release of triglycerides [10]. Under the skin, adipocytes are grouped together to form a more or less thick layer, which is generally referred to as subcutaneous adipose tissue or hypodermis. The amount of the fat mass depends on nutritional status: it increases with excessive calorie intake (obesity) and decreases with chronic nutritional deficiencies [11]. One of the most important key factors in this regard is the secretion by the adipose tissue of adipokines, which connect the adipose tissue with the pulmonary epithelia [12]. Some adipocytokines have cardioprotective, antiatherogenic and anti-inflammatory properties. One of the most interesting properties of adiponectin is that, unlike other adipokines that increase in obese patients, its expression in adipose tissue and its plasma concentration are reduced in overweight or obese patients. Adipose tissue is implicated in several lung diseases. In addition, an unbalanced diet plays a potential role in altering adipose tissue, also stimulating adipokine secretion, and also in the development and progression of lung cancer [13]. Indeed, it is a key mediator for several cancer-related processes, such as cancer cell proliferation, apoptosis, the regulation of tumor cell invasion and angiogenesis.

Recent studies have evidenced that nutrient deficiency may lead to the alteration of the immune system, as well as unintentional weight loss up to the state of cachexia that is linked to a worse prognosis in cancer [14]. The alteration of body composition, in particular muscle mass and fat and their relationship, may induce the negative prognostic condition called “sarcopenic obesity”, in which obesity and sarcopenia are present simultaneously, with a high fat mass and low muscle mass. Inflammation is the driving mechanism in the pathophysiology of cancer sarcopenia, and different inflammatory markers are related to sarcopenia, such as C-reactive protein (CRP) and the neutrophil-to lymphocyte ratio.

Baldessari et al. studied the correlation between body composition, nutrition and inflammatory status in NSCLC patients in order to improve the immune response against cancer; they analyzed several scores with different parameters in the most recent trials [15]. In cancer patients, the immune system and response to therapy are influenced by chronic inflammation and malnutrition. Malnutrition, in fact, impairs the ability of lymphocytes to proliferate and produce IFN-g [16]; in contrast, in obesity, several inflammatory cytokines are secreted by the macrophages M1 found in visceral adipose tissue [17] and T cells of adipose tissue [18]. Moreover, inflammatory status is also correlated to low levels of serum albumin with an increase in vascular permeability; therefore, hypoalbuminemia may be an indicator of the severity of inflammation [19].

This review aims to clarify the role of nutrition, with particular attention paid to anti-inflammatory and antioxidant foods for the prevention and/or treatment of lung cancer.

## 2. Oxidative Stress and Lung Cancer

Oxidative stress is one of the main determinants in the pathogenesis of many pathologies, such as aging and neurodegenerative pathologies, but above all cancer [20]. Oxidative mechanisms play a key role in the entire carcinogenesis process, namely initiation, promotion and progression [21]. Oxidative stress is mediated by free radicals, which are atoms or molecules that have at least one unpaired electron, and are therefore unstable and highly reactive towards other molecules [22]. It occurs when reactive oxygen species (ROS) and reactive nitrogen species (RNS) are not adequately removed or neutralized, or when the antioxidant defenses fail to metabolize them and exceed their capabilities [23]. The enzymatic antioxidant defense mechanisms against exposure to ROS are, respectively, superoxide dismutase (SOD), characterized by three isoforms [24], catalase (CAT), and finally glutathione peroxidase (GPx). These defense mechanisms can be altered by various factors such as nutrition, physical activity and aging [24]. There are also other types of non-enzymatic antioxidant defense that affect glutathione (GSH), ubiquinone Q 10, lactoferrin, flavonoids, carotenoids [25], and also micronutrients, such as manganese, iron, copper, zinc, selenium, chromium, etc. [26]. ROS are produced during major metabolic functions and represent the largest proportion of free radicals that are generated in living systems [27]. Mitochondria have always been considered as the main basis of intracellular ROS production, but in reality it can also be achieved by other enzyme systems such as NADPH oxidase or cytochrome P-450, cyclo-oxygenase and so on [27]. Furthermore, ROS and RNS can also be generated as a result of external stimuli such as inflammatory cytokines or growth factors, environmental factors, chemotherapy, ultraviolet rays (UV), ionizing radiation, and surgical interventions [25]. Under physiological conditions, ROS are in balance with the antioxidant defense system, which is essential for the survival of organisms and for their health [25]. However, this is not always the case. In some cases, ROS can act as anticarcinogens, for example, by promoting apoptosis or necrosis or by inhibiting angiogenesis thanks to their abilities [28]. At the same time, however, they can also have a carcinogenic effect, since the cells show metabolic disorders under oxidative stress or can damage the fundamental building blocks of their structure such as proteins, carbohydrates, DNA and RNA, or even lipid constituents of the membrane [29,30,31]. They can mediate cell damage by trying to couple their electrons to the target molecule, which in turn makes it unstable [23]. Carcinogenesis is a process that can be divided into several phases, initiation, promotion and subsequent progression, which determine the formation of malignant tumors [32,33]. During these phases, various genetic but also epigenetic events occur that lead to the progressive conversion of normal cells into cancer cells. ROS play a fundamental role, especially in the first phase, namely that of promotion, during which the gene expression of cells is modulated by influencing the genes that regulate cell differentiation and growth. The consequences of ROS activity on normal cells are mutations, chromosome aberrations, which lead to cell degeneration, carcinogenesis and aging [34]. In the progression phase, however, benign neoplasms are stimulated to grow faster and become malignant [35,36]. In addition, cancer-related inflammation is also linked to immunosuppression, which allows cancer cells to divert recognition by the immune system [37]. Indeed, inflammation resulting from the production of inflammatory cytokines and then ROS is a fundamental component of tumor progression.

Many tumors arise from sites of infection, resulting in chronic irritation and inflammation [38]. For example, due to its high carcinogenic potential and its synergistic effect with other respirable particles to generate ROS and to catalyze redox reactions in the human lung epithelium, cigarette smoking plays a crucial role in increasing the risk of epithelial inflammation and lung cancer, which leads to oxidative stress and an increased release of lung-derived inflammatory mediators [39,40,41,42,43]. Cigarette smoking has been shown to induce chronic airways inflammation with the accumulation and activation of leukocytes, which produce high levels of ROS and NO [44]. Furthermore, long-term exposure to cigarettes reduces the plasma concentrations of numerous antioxidants [45]. As for other substances, such as environmental pollutants, asbestos, polycyclic aromatic carbohydrates, arsenic and diesel emissions have been identified as potential causes of lung cancer [46], although their carcinogenic effects appear to be significantly lower than the harmful chemicals in tobacco smoking [47]. Many of these carcinogens also act covalently on DNA, causing oxidative damage that can induce DNA breaks [46]. Furthermore, studies show that, in reality, many air pollutants determine the activation of certain signaling pathways that could trigger pathological responses in the lung, such as the MAP kinase (MAPK) pathway that produces inflammation [48]. This could be a possible cause of lung cancer even in non-smokers [49].

## 3. The Importance of Adipose Tissue in Cancer Development and/or Progression

Adipose tissue (AT) is a complex organ that is important for the regulation of the body’s energy balance [50]. The primary function of AT is to store energy as lipids in adipocytes and to release them in response to physiological energy needs [51]. In addition to adipocytes, AT is made up of several other cell types within the stromal vascular fraction that have different functions. Mesenchymal stromal cells of AT, also called adipose stromal cells (ASC), are perivascular cells that support the endothelium and serve as adipocyte progenitors [52]. A heterogeneous palate of innate and adaptive immune cells, including macrophages, dendritic cells, mast cells, eosinophils, neutrophils, and T and B lymphocytes, are also contained in AT [53]. Taken together, adipocytes and other stromal cells of AT serve as a source of bioactive molecules that regulate important signaling pathways that are involved in cancer initiation and progression.

AT composition and physiology predetermine susceptibility to metabolic syndrome and its complications. Studies using mouse models have shown that, upon reaching a certain overgrowth threshold, AT becomes inflamed and fibrotic, and it has been suggested that AT dysfunction and chronic inflammation promote tumorigenesis and cancer progression [54]. Factors such as the anatomical location of AT, gender, age, and metabolic status can alter the tissue environment and characteristics in ways that we are only just beginning to understand [55]. A growing body of evidence indicates that AT accretion and deregulation in obesity, rather than the lifestyle responsible for obesity onset, is the key determinant of cancer initiation and progression [55]. In addition to storing energy, AT functions as an endocrine organ that secretes bioactive molecules termed adipokines [56]. To date, more than 50 different adipokines have been identified, the vast majority of which are produced by adipocytes [56]. In obesity, with an increase in AT mass and cellularity, circulating adipokine levels are altered. Leptin and adiponectin are the most thoroughly studied adipokines that are specifically produced by adipocytes. While the leptin level increases in obesity, the adiponectin level decreases, and it has been found that this altered leptin/adiponectin ratio correlates with cancer aggressiveness [57]. Other growth factors and cytokines implicated in the progression of obesity-related cancers include TNFα, IL-6 and IGF-1 [58]. Recently, a carcinogenic role for the AT-secreted chemokine CXCL12 (SDF1α) was discovered. Non-peptide AT-endocrine factors, including steroid hormones and lipids, also modulate processes, ranging from the remodeling of the extracellular matrix (ECM) to cancer cell signaling and metabolism [59]. Overall, adipokines promote tumor growth either though oncogenic signaling or through indirect mechanisms such as angiogenesis and immunomodulation [59]. Since the role of adipose tissue in cancer was first investigated, it was believed that all of the body’s AT depots stimulate cancer progression through the systematic circulation of these endocrine factors. However, the observation that cancers not surrounded by AT are not associated with obesity suggests that carcinomas are promoted by direct local exposure to proximal adipose cells. Adipocytes are typically absent from the normal parenchyma of epithelial glands. However, in invasive carcinomas, adipocytes come in direct contact with tumors, especially in the reproductive (prostate, uterus, breast) and digestive organs [60].

In obesity, an insufficient oxygen supply of hypertrophic adipocytes leads to cell death, which triggers a dynamic activation of innate and adaptive immune populations. Infiltrating and resident immune cells are potent sources of cytokines, proinflammatory chemokines, growth factors and matrix degradation enzymes, such as matrix metalloproteases (MMPs), which reshape tissues and induce low-grade chronic inflammation [61]. Cancer development is strongly linked to inflammation and, as a result, obesity. Tumors can be considered as wounds that "do not heal" due to the chronic inflammation induced by immune cells [62].

In AT, innate and adaptive immune cells make up almost half of the population of lipid-free cells in the stromovascular fraction. In healthy AT, immune cells maintain tissue homeostasis and an immunosuppressive microenvironment by eliminating apoptotic cells, regulating angiogenesis and reshaping the ECM [63]. In obesity, the ability of immune cells to perform these functions is often impaired, favoring inflammation, which is critical for the development of AT fibrosis [53]. Increased tissue stiffness and the mechanical transduction of fibrotic tissue can make an important contribution to tumor development [53].

AT plays a role in tumorigenesis, but not much is known about how adipose tissue is involved in cancer development. The adipokines produced by AT are not directly mutagenic, and for this reason it is assumed that the dysregulation of the AT can enhance mitogenic mechanisms in epithelial cells that already have cancer mutations [64]. The data from the literature show that DNA damage is mainly due to oxidative stress; obesity leads to increased ROS production and thus a greater risk of DNA damage, creating inflammation and therefore a microenvironment useful for cancer development [64]. An imbalance in the adipose tissue microenvironment (ATME) could therefore lead to inflammation and the subsequent development of cancer cells [65]. An imbalance in the microenvironment of adipose tissue leads to an increased production of inflammatory cytokines, such as TNFα, IFNγ, IL-1β, and IL-6 [66,67,68,69,70]. Indeed, other key molecules involved in the regulation of inflammation have recently been identified, such as the molecules of the WNT-5A family. A promotion of the inflammatory response in AT has been associated with this [71], as well as lipocalin 2 (LCN-2), a component of the immune system that plays a role primarily in the acute phase response of the infection, in particular in the induction of apoptosis, often mainly at the level of complete rest and the liver; it is also closely related to the phallus bifida and insulin resistance with increased levels, especially in obese and diabetic patients [72]. 

In addition, chitinase-3-like protein 1 (CHI3L1), also known as YKL 40, activates the innate immune system and carries out important functions in the inflammation of the tissues and in the remodeling of the extracellular matrix. This protein is remarkable as it is a growth factor with key functions in cancer development, especially that of the colon, which is associated with obesity [73,74]. Circulating YKL 40 levels are strongly linked to high levels of C-reactive protein and IL-6 [74,75]. There is, therefore, increasing evidence that obesity is clearly linked to an overall risk of cancer development and progression through adipose tissue inflammation [65]. As can be seen, changes in ATM affect the biology of the tumor, with particular attention paid to the types of tumors that are exposed to direct increases in AT [65]. The study of the modulation of the altered secretion of adipokines and the dysfunction of the adipose tissue organ could therefore represent a new therapeutic goal in the treatment of obesity as well as related pathologies [76].

## 4. Adiponectin and Lung Cancer

Obesity is one of the risk factors associated with the incidence and progression of multiple cancer types, although the molecular and cellular mechanisms by which adipose tissue affects both tumor initiation and progression are not fully understood. Adipocytes play an important role in tumor growth; they actually produce and secrete various adipokines to facilitate interorgan crosstalk and indirectly affect tumor cell biology by regulating insulin resistance and inflammation. Changes in the adipokine system likely interfere with interorgan crosstalk in lung cancer, which can affect the lung tumor microenvironment. Not all adipokines stimulate tumor growth; in fact, adiponectin (Acrp30) is an important adipokine with anti-inflammatory and beneficial metabolic effects, and is a protein hormone with 244 amino acids that signals through three distinct receptors, AdipoR1 and AdipoR2 (expressed in several organs, tissues, and cell lines) and a third receptor protein, T-cadherin (a receptor mainly expressed in the vascular system). Acrp30 exists as oligomers with low (LMW), medium (MMW), and high molecular weights (HMW), the latter form having the most important biological effects [77]. Several datasets support the hypothesis of both direct and indirect roles for Acrp30 as a regulatory mediator of various mechanisms underlying lung carcinogenesis. From a molecular point of view, Acrp30 inhibits carcinogenesis by regulating both cell growth and the levels of inflammatory cytokines. The expression of adiponectin is deregulated in several types of cancer [78]. Studies on human lung adenocarcinoma A549 cells have shown that adiponectin can inhibit the CREB transcription factor and induce the cell cycle in these lung cancer cells [79]. It has been shown that, in A549 cells, Acrp30 causes, in a time- and dose-dependent manner, a reduction in cell viability, and increases the cell apoptosis rate and lipid peroxidation, while at the same time reducing the release of nitric oxide, both markers of cellular oxidative stress [80]. In lung tissue samples from NSCLC, the reduced expression of adiponectin is related to the development of NSCLC through the negative regulation of MMP-9 expression [81]. In a recent study, Nigro et al. investigated the concentration of Acrp30 and its receptors in serum and tissue samples from NSCLC patients. They found a significant reduction in the total serum levels and expression of Acrp30 in NSCLC patients compared to normal subjects. Of the three types of Acrp30 oligomers, the HMW are the most downregulated. They also observed a significantly higher expression of AdipoR1, no differences in R2 and a lower expression of T-cadherin in lung cancer samples compared to normal healthy lung tissues [82]. Overall, these studies have underscored the important role of Acpr30 in both the development and progression of lung cancer by inducing the inhibition of both processes. A study conducted on UK Biobank shows that diets high in fruits, vegetables, breakfast cereals, and dietary fiber, with a low intake of red meat and processed meat, are associated with a lower risk of lung cancer [83]. A long-term ketogenic diet increases serum adiponectin levels and does not affect serum IGF-1 levels [84].

**Figure 1:**
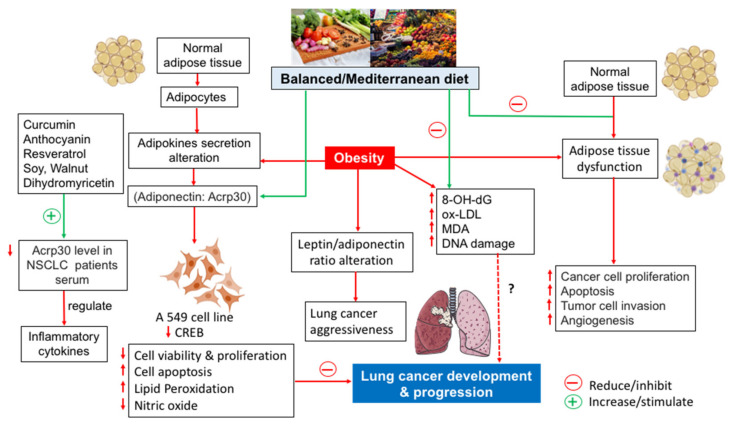
Balanced diet effects on the prevention of lung cancer mediated by adipose tissue dysfunction.

Various studies have demonstrated that the addition of medicinal plants and herbal bioactive compounds, especially curcumin, anthocyanins, resveratrol, soy, walnut, and dihydromyricetin, can be used to increase plasma adiponectin [85], and this can be useful in the prevention and treatment of lung cancer (Figure 1). The modulation of Acrp30 can affect the microenvironment of the lung tumor and the use of nutritional factors that can increase the concentration of Acrp30 could be a new therapeutic strategy for contrasting lung cancer.

## 5. Anti-Inflammatory and Antioxidant Foods to Prevent Cancer

In recent studies, a better adherence to the Mediterranean diet has been linked to a lower risk of cardiovascular disease, diabetes, cancer, neurodegenerative pathology [86,87] and a lower mortality rate [88]. The reason for these beneficial effects is the capacity of the Mediterranean diet to reduce oxidative stress thanks to the high intake of antioxidants [89,90]. As the basis of the Mediterranean diet, we can mainly find fruits and vegetables, cereals, legumes, nuts and seeds; a moderate intake of dairy products, poultry, preferably blue fish, eggs and wine; and finally, a low intake of red meat. Olive oil, on the other hand, is thought to be the main source of fat [91]. The antioxidant properties of the Mediterranean diet could be attributed to the detoxification mechanisms of ROS [92], the decrease in the -Oxo-2′-deoxyguanosine (8-OH-dG) levels and the low interaction of ROS with DNA [93]. Recent studies have shown that the Mediterranean diet is associated with low levels of oxidative stress biomarkers, such as ox-LDL [94] and malondialdehyde (MDA) [95]. Indeed, it is known that the stability of DNA molecules is essential for the maintenance of normal cell functions and damaged DNA could promote the occurrence of a number of acute and chronic diseases [96,97]. A balanced diet is, therefore, the fundamental cornerstone of our antioxidant defenses, thanks to the presence of many food constituents that prevent antioxidants from working optimally [98], as well as being a key factor in the prevention of many diseases such as obesity [99,100].

It is accompanied by mild inflammation, which is characterized by an increase in the production of proinflammatory cytokines and proinflammatory adipokines, IL-β, IL-6 and TNF α, caused by the cells of white adipose tissue (WAT), as well as by the infiltration of inflammatory cells in the same adipose tissue [101,102]. It is widely believed that a diet rich in fruits and vegetables plays a key role in the prevention of many human diseases [103]. Plants are rich in antioxidants and provide most of those found in the human diet [104].

Phenols, for example, are compounds with an -OH group attached to a benzene ring and defined as excellent scavengers of different ROS. Some studies by Romagnolo and Selmin [105] have highlighted how food flavonoids exert protective effects against different types of tumors, including lung [106], gastric, hepatic, prostate, and ovarian tumors, etc., through their protective effects against the damage to DNA caused by free radicals [107], presumably through the chelation of metal ions. Flavonoids complexed with copper or iron prevent the generation of ROS [108]. Phenols can be found in a variety of foods, such as olive oil, whole grains, fruit, vegetables, nuts, tea, coffee and red wine [109,110,111,112].

In fact, some studies have found that some phenols stimulate the secretion of adiponectin and the subsequent activation of AMPK, which are directly linked to the inhibition of NF-κB and macrophage infiltration, with a consequent reduction in the formation of new adipocytes and anti-inflammatory effects on AT in vitro and in obese animals [113,114,115]. In recent years, mainly contained in EVOO oil, not only the beneficial effects of its daily consumption have been shown, but also how hydroxytyrosol and its derivative oleuropein inhibit the oxidation of LDL in vitro and prevent many metabolic disorders, including obesity [116,117,118,119]. Spices or red wine are particularly rich in polyphenols, such as rosmarinic acid, and, if added to meat [120] before cooking, could have a beneficial effect by reducing the formation or absorption by the gastrointestinal tract of MDA, an important active compound in cytotoxic lipogenesis. Elevated MDA levels are associated with an increased risk of atherogenesis, cancer and lipid peroxidation [121,122]. Furthermore, the microbiome could be influence the metabolism and the effects of polyphenols, especially isoflavoids [123]. In addition, among the compounds with anti-inflammatory, anti-tumor and immunomodulating anti-microbial activities [124], we find propolis. These functions are attributed to flavonoids, and some phenolic acids contained in the compound propolis exert immunomodulatory effects on a wide range of immune cells, which are mediated by the modulation of the signaling pathways of kinase 2 and MAPK and regulated by extracellular signals. Propolis also modulates the nuclear factor of activated T cells and the NFKB signaling pathway and stimulates antibody production, suggesting that it could be used as an adjuvant in the inflammatory stages [124]. Among the phenylpropanoids, we also find cinnamal aldehyde, an organic compound that is found in abundance in cinnamon essential oils. This is generated through the inflammation induced by TNFα, through the suppression of the activation of NF-_K_B [125]. Among spices, we also find piperine, which is present in black pepper [126]. Piperine has powerful anti-inflammatory functions by regulating prostaglandins through the inhibition of pro-inflammatory cytokines such as IL-6 [126]. Piperine promotes innate immunity by increasing the activity of phagocytes, and consequently, that of microglial cells [127]. Other compounds that are essential for their antioxidant and anti-inflammatory capabilities are carotenoids [128]; in particular, beta-carotene is principally considered a precursor of vitamin A. Foods that are rich in carotenoids are fruits, vegetables, cereals, coffee and wine, which have shown remarkable antioxidant activity in vitro, as revealed by a series of test-tube tests of the total antioxidant activity such as the Oxygen Radical Absorbance Capacity (ORAC), ABTS [129].

One of the characteristics of carotenoids is their conjugated double bonds, which allow these components to accept electrons from reactive species and thus neutralize ROS [130]. A combination of lipophilic antioxidants such as vitamin E or vitamin C with β-carotene could lead to significantly greater beneficial effects than those of a single antioxidant [131]. Studies have shown that β-carotene reduces the expression of the heme oxygenase-1 gene in human dermal fibroblast (FEK4) cells exposed to ultraviolet radiation [132,133]. Another carotenoid that is mainly found in tomatoes [134] is lycopene. It is known to be a powerful antioxidant against ROS generated by cigarette smoke and to modulate redox-sensitive cellular targets such as protein kinase, protein tyrosine phosphatase (PTP), MAPK and transcription factors [135].

Another medicinal and bioactive constituent is curcumin, well known for its anti-inflammatory and antioxidant properties as well as its high reactivity to peroxyl radicals. The curcumin extracted from turmeric could act as a chemotherapeutic agent and have a preventive effect against, for example, tumors of the colon, the skin of the oral cavity or the intestine [136]. It also inhibits the production of the pro-inflammatory cytokines IL-6 and TNFα in lipopolysaccharide-stimulated BV2 microglial cells (LPS) [137]. Curcumin inhibits the cyclooxygenase 2 (COX-2) and STAT signaling pathways [138]. It also works as a free radical scavenger and increases the production of antioxidant enzymes [139,140]. The antioxidant and anti-inflammatory effects are determined by the role of many vitamins or mineral salts present in a variety of foods [141]. Vitamin C is an essential cofactor for the action of numerous enzymes [142]. It is able to directly eliminate various ROS [142], and this role could be especially important in the respiratory tract, where it helps to absorb inhaled and highly oxidizing ROS, such as ozone and nitrogen dioxide, which often pollute the air we breathe, by eliminating many free radicals in cigarette smoke; hence, smokers consume vitamin C faster than non-smokers [143]. In addition, due to its role in the immune system, vitamin C can protect against infections [144]. Various spices, herbs, fruits and vegetables have proven to be excellent sources of vitamin C [145]. For example, thyme, turmeric, coriander and beetroot juice are excellent sources of vitamin C [146]. Vitamin E is also an important antioxidant molecule, especially in relation to alpha-tocopherol, and many studies analyzing its fundamental effects have been conducted, especially on the brain and nervous system, aiming for the prevention of neurodegenerative diseases [147,148]. Tocopherols are mainly found in edible oils, such as corn, soy, sesame and rapeseed (canola) oils, or in walnuts [149]. Tocotrienols are present in very small amounts in oils derived from rice bran, barley, and wheat germ [149]. A lower vitamin E intake has been associated with an increased risk of cancer [150]. However, the findings are still controversial. The γ and δ forms of tocopherols and tocotrienols (T3) appear to have much lower systemic bioavailability compared to α-T, but at the same time show a superior cancer preventive activity compared to alpha-tocopherol in many studies on animal models and cell lines [150].

Among the mineral salts with strong antioxidant or anti-inflammatory effects, we find selenium [151]. It is naturally abundant in foods such as corn, garlic, onion, cabbage and broccoli [152]. It is an essential component that plays a role in various physiological processes, particularly at the level of the immune system [151]. Studies have shown that selenium supplementation modulates the inflammatory response in patients with respiratory distress syndrome by restoring the antioxidant status of the lungs and reducing levels of the pro-inflammatory cytokines ILβ and IL-6 [153]. In addition, supplementation with selenium demonstrates an improvement in the TCD + lymphocyte count [154] and improves glutathione peroxidase and other antioxidant selenoenzymes together with the activities of catalase [155]. Overall, through its non-enzymatic role, selenium improves immunity by acting as a cofactor for the enzymes involved in the post-translational modifications of proteins [151].

Other important nutritional components of the traditional Mediterranean diet are ω-3 fatty acids, which have anti-inflammatory properties [156]. They are mainly found in fresh fish, rapeseed (canola), vegetable oils and nuts, and are considered to be important longevity factors [156]. In particular, eicosapentaenoic acid (EPA) and docosahexaenoic acid (DHA) induce a cardioprotective response by modulating the structure and fluidity of the cell membrane, thereby improving cardiac mitochondrial functions and energy production. Consequently, their presence influences the action of membrane-bound enzymes and receptors [157,158]. On the other hand, n-3 PUFAs counteract the release of proinflammatory cytokines both in vascular tissue and in the myocardium and promote a better vascular reactivity and myocardial performance [159], and are found in products of animal origin, especially fish. Researchers paid particular attention to the possible neuroprotective effect of astaxanthin [160]. This ketocarotenoid is produced by one species in particular (Haematoccocus pluvialis) and other microalgae and is responsible for the reddish-orange coloration of some fish such as salmon, shrimp and lobster. According to a study by Galasso et al. [161], astaxanthin crosses the blood–brain barrier and has effects on the central nervous system, with anti-inflammatory and anti-apoptotic properties, and is promising for future therapeutic applications against Alzheimer’s disease [161].

As for milk and its derivatives, it has been found that fermented dairy products are associated with the modulation of the inflammatory and immune responses caused by the presence of bacteria in dairy products and their metabolites, e.g., conjugated linoleic acid (CLA), the different fractions of casein with antioxidant effects, lactoferrin, bioactive peptides, etc. [162,163]. Probiotics exert their anti-inflammatory and immunomodulatory effects by regulating the NF-_K_B and MAPK signaling pathways [164], as well as the vitamin D present in dairy products [165]. It increases the antioxidant level of NRF-2 and facilitates mitochondrial functions, lipid peroxidation and DNA damage [166]. Milk also contains a high level of palmitic acid, which is known to act selectively on Toll-like receptors and trigger reactions of the innate immune system [167]. In addition, fatty acids, which are essential for maintaining intestinal health and the microbiome, could reduce the production of proinflammatory cytokines by macrophages [168]. Eggs also appear to have antioxidant activity. In addition to the yolk, which contains a minimal amount of carotenoids, the albumen seems to be the part of the egg that is most involved in these mechanisms [169]. It is richer in proteins; these proteins as well as the peptides derived from their enzymatic hydrolysis have been recognized for their functional importance due to their antioxidant, anti-microbial, metal-chelating, and anti-tumor activities, but above all as angiotensin-converting enzyme inhibitors (ACE) [170]. However, these peptides are still only known for their beneficial in vitro activities. Further studies are currently required to prove their importance in vivo as well [169].

**Figure 2:**
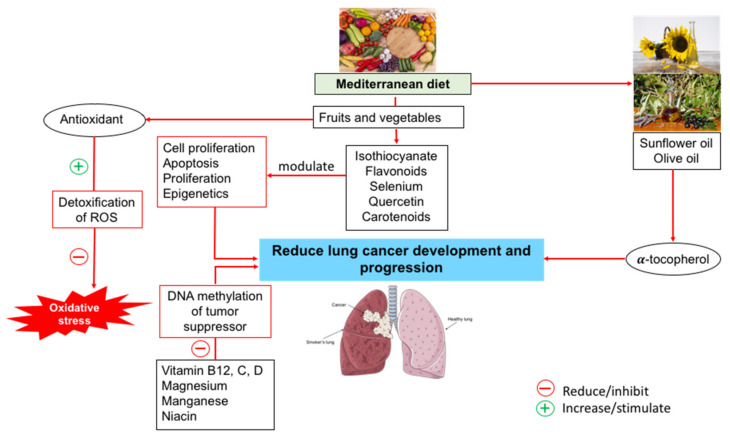
The Mediterranean food and lung cancer development and progression.

Therefore, it is evident that numerous components of the Mediterranean diet have beneficial antioxidant and anti-inflammatory effects, underscoring the possibility of improving chronic stress and inflammation, as well as the possible reduction in the incidence of cancer (Figure 2).

## 6. Foods and Lung Cancer

The survival rate of lung operated patients is between 5 and 16% [171]. Treatment modalities used to treat lung cancer include chemotherapy and radiation, which can increase oxidative stress [171]. The treatment of the cancer depends on the histological type, the medical comorbidities and the prognostic indicators of the disease stage, weight loss and the success of the operation [172,173,174]. Radiation therapy in particular is associated with the development of significant acute toxicities such as esophagitis, dysphagia, anorexia and fatigue [175,176]. Additionally, cancer patients treated with chemotherapy are more likely to experience nausea, vomiting and hair loss [175]. These symptoms can impair the ability to achieve adequate nutritional intake and consequently increase the risk of weight loss and developing malnutrition [177]. Furthermore, it is now common practice to reserve parenteral nutrition for patients with a non-functioning gastrointestinal tract [178]. Malnutrition is associated with negative clinical outcomes in cancer patients, including a decreased quality of life [179,180,181], reduced nutritional status [180,181,182,183], increased use of health care [184] and poorer survival [185,186].

In recent years, there have been numerous studies aimed at understanding whether nutritional interventions that improve patients’ nutritional status have a greater impact on patients’ quality of life, i.e., on the response to treatment and survival [187]. Early studies of nutritional interventions in lung cancer patients have shown mixed results [188,189,190]. A recent randomized pilot study has demonstrated the feasibility of investigating intensive, individualized dietary counseling in lung cancer patients receiving radiation therapy, paving the way for future larger studies to determine the effectiveness of these interventions. This study showed clinically important differences in favor of the intervention for weight, lean mass, physical well-being and functional well-being, but these results require confirmation in a larger sample [191]. The role of nutrition is also becoming important for the outcome of lung cancer surgery, as malnutrition is believed to increase the risk of postoperative complications [192]. Some nutrients and phytochemicals have been shown to have anti-inflammatory and antioxidant properties that can directly affect or prevent pathogenesis [193]. Thus, nutrition proves to be an elementary component in the primary prevention of lung diseases [194], since in both cases it reduces the oxidative damage to DNA and thus protects against cancer [195]. Nutrition and antioxidants could affect oxidative stress levels [195]. The Mediterranean diet has been shown to have a protective role in the prevention of non-communicable diseases and is well established [196]. There is considerable evidence that high adherence to the Mediterranean diet lowers the risk of various types of cancer [197], which is why the Mediterranean diet remains one of the most complete nutritional patterns in terms of macro- and micronutrients [197].

The Mediterranean diet is a predominantly plant-based diet with beneficial effects on health. In 2010, it was included in the list of the intangible cultural heritage of humanity by the United Nations Educational, Scientific and Cultural Organization [198]. Fruits and vegetables are known to contain numerous antioxidants, fiber, minerals and phytochemicals that can help prevent cancer and contribute to a healthy weight [199]. Plant foods contain thousands of biologically active phytochemicals, such as isothiocyanate and flavonoids. Many of these compounds inhibit the phase-one enzymes that metabolize carcinogens, induce phase-two detoxification enzymes, improve the immune system and modulate the levels of circulating hormones [200]. Evidence from cohort studies, case–control studies, prospective studies and a systematic review article shows that individuals with a high dietary intake of fruit or vegetables have a lower risk of lung cancer than those with a low intake of fruit and vegetables [201,202]. There is limited evidence that non-starchy vegetables and foods containing selenium and quercetin lower the risk of lung cancer [203]. A Danish cohort study carried out from 1993 to 2001 observed an inverse relationship between the high intake of plant foods, fruits, vegetables, legumes and potatoes and lung cancer [201]. An earlier cohort study in Finland from 1985 to 1993 showed the same results and in particular how a diet rich in carotenoids can reduce the risk of lung cancer [204]. Additionally, a previous expert’s report by the World Cancer Research Fund/American Institute for Cancer Research did not mention the differences between different histological subtypes of lung cancer in terms of the effect of fruit and vegetable consumption [203].

These studies have suggested that antioxidants from fruit and vegetables strongly reduce the oxidative stress caused by smoking, while others have found that fruits offer more protection to non-smokers. Among the various micronutrients, particular attention should be paid to some of the isoforms of vitamin E found in vegetable oils. Sunflower oil has been found to play a protective role, but both vegetable and animal butters could play a detrimental role in lung cancer [19]. Compared to other vegetable oils, sunflower oil has a low content of gamma-tocopherol (an isoform of vitamin E) [205], but is enriched with alpha-tocopherol. A recent study showed that gamma-tocopherol can promote lung cancer [206], while alpha-tocopherol, found in olive and sunflower oils, reduces the risk of this disease. Recent studies have also observed that phytoestrogens and glucosinolate hydrolysis products could also be other potential micronutrients in fruits and vegetables that may prevent lung carcinogenesis [207,208]. Hence, it can be concluded that the use of dietary antioxidants and nutritional supplements of vitamins and minerals in cancer patients reduces inflammation and infection [209], cancer growth [210], mutation [211], and the proliferation of malignant cells, and induces differentiation [212] and de-differentiation [213]. Furthermore, this supplementation has been associated with pain relief [214,215].

It was recently discovered that gene methylation in sputum was also strongly associated with a decrease in lung function. Several nutrients, for example, vitamin B, vitamin C [216,217], and phytochemicals [218,219], have been shown to have effects with regard to modulating epigenetics, so dietary interventions are therefore promising for the chemoprevention of lung cancer [220]. Folate is a typical methyl donor involved in carbon metabolism. Interestingly, it was suggested that folate deficiency can lead to the methylation of the tumor suppressor gene promoter [221]. In a cohort of smokers, folate intake showed a beneficial effect on the maintenance of lung function [222]. In fact, five additional factors have been identified, including vitamins D and B12, manganese, magnesium, and niacin as protective agents against gene methylation [223]. The availability of folate and vitamin B12 is a limiting factor for global DNA methylation. As for the fat component, diets with anti-inflammatory components such as Ω-3 fatty acids appear to be promising, since inflammation plays an important role in the pathogenesis of cancer [220]. In some animal models, a high-fat diet has been associated with chromatin changes [224,225]. So far, it has been shown that short-chain fatty acids are associated with the inhibition of the histone deacetylases (HDAC) in vitro [226]. In addition to short-chain fatty acids, dietary Ω-3s have also demonstrated inhibitory properties of a certain Enhancer of the Zeste 2 Polycomb Repressive Complex 2 Subunit (EZH2) in cancer cells [220]. Conversely, the consumption of red and processed meats plays a critical role in carcinogenesis [227]. The fat content of red meat, carcinogenic products resulting from culture, high-temperature storage and the endogenous formation of mutagens in meat are associated with the pathway of carcinogenesis [228,229]. In fact, heterocyclic amines (HCAs) and polycyclic aromatic hydrocarbons (PAHs), the main by-products of the cooking process, exert carcinogenic effects, especially at high temperatures [230,231]. In addition, red meat was recently found to be linked to cancer induction as a source of saturated fat and iron [232]. Tasevska et al. observed a moderate positive association between meat consumption and lung cancer [19]. Fish is not a risk factor for lung cancer; on the contrary, it contains omega 3, which is beneficial in this regard [19]. In a study by Brennan et al., it was shown in cases of non-smokers that the consumption of liver and eggs is not associated with the development of lung cancer [233]. As for the potential risk of dairy products for lung cancer, this may be due to the fact that they are the main source of calcium. Indeed, a recent meta-analysis found a positive association between diets rich in calcium and the risk of developing cancer, but the results are yet to be confirmed. In addition, the presence of lactic acid bacteria in dairy products and some types of cheese, as well as their interaction with the immune system, could influence cancer induction [234]. Furthermore, it has also been shown that a ketogenic diet can improve responses to radiation therapy and chemotherapy in cancer cells through a mechanism that involves oxidative stress [235]. As observed, compared to normal cells, cancer cells are able to survive in a state of chronic metabolic oxidative stress, which is characterized by particular levels of O_2_ and H_2_O_2_. This increase in the ROS levels in cancer cells can be compensated for by the increase in glucose metabolism via the pentose phosphate pathway, which leads to the generation of NADPH, which is used as a cofactor in hydroperoxide metabolism [236]. First, since ketogenic diets force cells to rely on mitochondrial oxidative metabolism for energy by limiting the availability of glucose, a ketotic state or ketosis would be expected to exacerbate metabolic oxidative stress in cancer cells compared to normal cells. Second, due to the inhibition of insulin, hepatic ketogenesis increases the blood levels of the ketone bodies beta-hydroxybutyrate and acetoacetate, both of which have been shown to inhibit histone deacetylase at the cellular level. HDAC inhibitors are known to reduce tumor cell proliferation and increase apoptosis [237,238].

There are not many studies that have reported the role of nutrition associated with a specific lung cancer treatment. Nevertheless, it is recognized the important role of nutrition in lung cancer prevention and/or treatment, given the potential anti-inflammatory and anti-oxidative effects of functional foods of correct nutrition in vivo and in vitro studies. Indeed, as reported Kiss et al, data literature showed that simple dietary counseling improves energy and protein intake during chemotherapy in patients with lung cancer [191]. In addition, the state of malnutrition of the patients undergoing this type of therapy is well known, for this reason nutrition plays a fundamental role.

## 7. Conclusions

This review analyses the important role of nutrition in the prevention and treatment of lung cancer. A large variety of dietary products possess chemopreventive and anti-tumor potential, acting to increase adiponectin levels and reduce inflammation and oxidative stress. These products could be promising candidates to prevent and treat lung cancer. Taken together, this review suggests that the regular food intake rich with the above-mentioned anti-inflammatory and antioxidant foods would reduce the incidence of lung cancer.
